# Hypoxia potentiates Notch signaling in breast cancer leading to decreased E-cadherin expression and increased cell migration and invasion

**DOI:** 10.1038/sj.bjc.6605486

**Published:** 2009-12-15

**Authors:** J Chen, N Imanaka, J Chen, J D Griffin

**Affiliations:** 1 Department of Medical Oncology, Dana-Farber Cancer Institute, Boston, MA 02115, USA

**Keywords:** Notch, hypoxia, hypoxia inducible factor (HIF), breast cancer, epithelial-to-mesenchymal transition (EMT)

## Abstract

**Background:**

Epithelial-to-mesenchymal transition (EMT) is associated with decreased adhesion and acquisition of metastatic potential of breast cancer cells. Epithelial-to-mesenchymal transition is mediated, in part, by two transcription repressors, Snail and Slug, that are known to be targets of the Notch signaling pathway, and JAGGED1-induced Notch activation increases EMT. However, the events that lead to increased Notch activity during EMT of breast cancer cells are unknown.

**Methods:**

The accumulation of hypoxia inducible factors (HIFs) under hypoxia was detected by western blot analysis, and their effects on Notch signaling were measured by an *in vitro* Notch reporter assay. The expression of Notch target genes under hypoxia was tested by real-time PCR. The knockdown of HIF-1*α* was mediated by retroviral delivery of shRNA. The expression of *Slug* and *Snail* under hypoxia was measured by real-time PCR. Breast cancer cell migration and invasion under hypoxia were tested with cell migration and invasion kits.

**Results:**

Hypoxia increased the expression of Notch target genes such as *HES1* and *HEY1* in breast cancer cells, as was expression of Notch receptors and ligands. The mechanism is likely to involve the accumulation of HIF-1*α* and HIF-2*α* in these cells by hypoxia, which synergised with the Notch co-activator MAML1 in potentiating Notch activity. Hypoxia inducible factor-1*α* was found to bind to *HES1* promoter under hypoxia. Knockdown of *HIF-1α* with shRNA inhibited both *HES1* and *HEY1* expression under hypoxia. Hypoxia increased the expression of *Slug* and *Snail*, and decreased the expression of *E-cadherin*, hallmarks of EMT. Notch pathway inhibition abrogated the hypoxia-mediated increase in *Slug* and *Snail* expression, as well as decreased breast cancer cell migration and invasion.

**Conclusion:**

Hypoxia-mediated Notch signaling may have an important role in the initiation of EMT and subsequent potential for breast cancer metastasis.

Notch signaling impinges on a wide variety of cellular processes, including cell fate specification, cell proliferation, differentiation, apoptosis and the maintenance of stem cells. Recent studies suggested an important role for the Notch pathway in breast cancer. *Notch1* and *Notch4* were identified as mouse mammary tumour virus (MMTV) integration sites in murine mammary tumours (Smith *et al*, 1995; Gallahan and Callahan, 1997; Dievart *et al*, 1999). Transgenic mice harbouring constitutively active Notch4 intracellular domain under the control of either *MMTV* promoter (Smith *et al*, 1995) or whey acidic protein (WAP) promoter (Gallahan *et al*, 1996) showed arrested mammary gland development and eventually developed poorly differentiated adenocarcinomas. Mammary-specific over-expression of constitutively active Notch1 or Notch3 intracellular domain led to the formation of aggressive, metastatic breast tumours (Kiaris *et al*, 2004; Hu *et al*, 2006). *c-Myc* was found to be a direct transcriptional target of aberrant Notch1 signaling and was required for Notch1-induced murine mammary tumourigenesis (Klinakis *et al*, 2006). Recent studies also pointed to a role for Notch signaling in human breast cancer. The expression of all four Notch receptors has been reported in human breast cancer at varying frequencies (Callahan and Egan, 2004), and the expression of Notch ligands such as JAGGED1 correlated with a more aggressive disease course (Reedijk *et al*, 2005). JAGGED1-mediated Notch activation induced epithelial-to-mesenchymal transition (EMT) (Leong *et al*, 2007). Furthermore, low levels of the Notch antagonist Numb was found to correlate with both high Notch signaling and sensitivity to a *γ*-secretase inhibitor (Pece *et al*, 2004). NOTCH1 has also been implicated as a downstream effector of oncogenic Ras in human mammary tumourigenesis (Weijzen *et al*, 2002). Recent studies also suggested an important oncogenic collaboration between Wnt and Notch pathways in breast tumourigenesis. Ectopic Wnt-1 expression transformed human mammary epithelial cells through a mechanism that requires an increase in Notch signaling (Ayyanan *et al*, 2006).

Solid tumours often have low oxygen tension environments because of insufficient and defective vascularisation. Tumour hypoxia is associated with enhanced invasiveness, angiogenesis and distant metastasis. Hypoxia inducible factors (HIFs) are heterodimeric proteins belonging to the basic helix-loop-helix transcription factor families. Hypoxia inducible factor-1*α* is over-expressed in many solid tumours, including breast cancer (Semenza, 2003). Stabilisation and activation of HIF-1*α* transcription complex also correlates with tumour metastasis and poor prognosis in cancer patients (Harris, 2002; Semenza, 2002; Gupta and Massague, 2006). Culture of lung cancer cells (Chen *et al*, 2007) or ovarian cancer cells (Sahlgren *et al*, 2008) under hypoxia increased Notch pathway activation. Low oxygen content also potentiated Notch signaling in melanocytes through stabilisation of HIF-1*α* (Bedogni *et al*, 2008).

Epithelial-to-mesenchymal transition is one of the key mechanisms that induce tumour invasion and metastasis (Thiery, 2002; Thiery and Sleeman, 2006). Downregulation of E-cadherin is one of the best markers of EMT in human breast cancer (Vincent-Salomon and Thiery, 2003). Snail and Slug are two known E-cadherin repressors that are thought to initiate EMT in breast cancer (Martin *et al*, 2005). Upregulation of Snail correlates with metastasis and poor prognosis, whereas silencing of Snail is critical for reducing tumour growth and invasiveness (Perl *et al*, 1998; Moody *et al*, 2005). Slug has also been shown to induce EMT and metastasis through the repression of E-cadherin (Peinado *et al*, 2007). The expression of Slug and Snail has been shown to be regulated by Notch pathway (Leong *et al*, 2007; Sahlgren *et al*, 2008).

In this study, we show that Notch signaling is potentiated in human breast cancer cells by hypoxia. The mechanism is likely to involve accumulation of both HIF-1*α* and HIF-2*α* under low oxygen concentration, which then synergise with the Notch co-activator MAML1 in potentiating Notch activity in an *in vitro* Notch reporter assay. Chromatin immunoprecipitation (CHIP) experiments showed that, with hypoxia, HIF-1*α* bound to human *HES1* promoter. shRNA-mediated knockdown of *HIF-1α* inhibited hypoxia-induced *HES1* and *HEY1* expression, indicating the effect of hypoxia on Notch signaling is via HIF transcription factors. The expression of *Slug* and *Snail* was increased in breast cancer cells with hypoxia, which in turn suppressed the expression of *E-cadherin*. Finally, we found that Notch activation is required for hypoxia-mediated breast cancer cell migration and invasion.

## Materials and methods

### Plasmids and antibodies

pcDNA3/HIF-1*α* (P402A/P564A) and pcDNA3/HIF-2*α* (P405A/P531A) constructs were described earlier (Yan *et al*, 2007). MAML1 full-length, dominant negative MAML1 (a.a. 13–74), hemagglutinin (HA)-tagged murine ICN3 were described earlier (Wu *et al*, 2000; Weng *et al*, 2003, 2004). pRL-TK (Promega) encodes *Renilla* luciferase under the control of thymidine kinase (TK) promoter and was used to normalise firefly luciferase activities for transfection efficiency. *HES1*-luc contains the −194 to +160 human *HES1* promoter sequence that was cloned upstream of the firefly luciferase gene in the pGL2 basic vector (Promega, Madison, WI, USA). Hypoxia inducible factor-1*α* shRNA constructs (TG320380) were from OriGene (Rockville, MD, USA). mHes1 antibody was a gift from Dr Tetsuo Sudo. Notch1 antibody (C-20-R), Notch3 antibody (M-134), Notch4 antibody (H-225), Jagged1 antibody (C-20), Maml1 antibody N-20) and Slug antibody (D-19) were from Santa Cruz Biotechnology Inc. (Santa Cruz, CA, USA), Notch2 antibody (C651.6DbHN) was from Developmental Studies Hybridoma Bank at the University of Iowa. Jagged2 antibody was from Cell Signaling (Danvers, MA, USA). Hypoxia inducible factor-1*α* antibody was from Novus (Littleton, CO, USA). Hypoxia inducible factor-2*α* antibody was a gift from Dr Yoji Minamishima and Dr William Kaelin Jr. E-cadherin antibody was from BD Biosciences (San Jose, CA, USA). *β*-actin antibody was from Sigma-Aldrich (Saint Louis, MO, USA).

### Cell culture and transient transfection

Human osteosarcoma cell line U2OS, human breast cancer cell lines MDA-468, MDA-231, MCF7, T47D, SK-BR-3 and ZR-75–1 were purchased from ATCC (Manassas, VA, USA) and were cultured in Dulbecco's modified Eagle's medium (DMEM) containing 10% fetal bovine serum. Transient transfections were carried out using Lipofectamine 2000 transfection reagent (Invitrogen, Carlsbad, CA, USA) according to the manufacturer's instructions.

### Real-time PCR

Total RNAs were isolated with the Trizol (Invitrogen) method, and cDNAs were synthesised with SuperScript III first-strand synthesis system (Invitrogen) from 1 *μ*g of total RNA. Real-time PCR was performed with the cDNA samples and 2 × SYBR PCR master mixture (PE Applied Biosystems, Foster City, CA, USA) using an ABI PRISM 7500 sequence detector (PE Applied Biosystems) and the formation of PCR products were monitored using the SYBR green method (PE Applied Biosystems). All samples were amplified in duplicates. The relative changes in the amount of transcripts in each sample were determined by normalising with the *β*-actin mRNA levels. The sequences of the primers used in real-time PCR are listed in Supplementary Table 1.

### Notch reporter assay

U2OS cells were plated at 5 × 10^5^ cells per well in the six-well plates and were transfected the following day with cDNA constructs as indicated in the figure legends using the Lipofectamine 2000 transfection reagent (Invitrogen). The total amounts of plasmid DNA were kept constant by adding appropriate amounts of empty vectors. Cell lysates were made at approximately 48 h post-transfection and the luciferase activities were measured with the dual luciferase reporter assay systems (Promega). Relative luciferase activities were determined by normalising *HES1*-luc firefly luciferase activity with *Renilla* luciferase activity.

### Western blot analysis

Human breast cancer cells were cultured under 21% O_2_ or 1% O_2_ conditions for the indicated time and cells were washed with ice-cold PBS and were lysed *in situ* with a solution containing Tris (20 mM, pH 8.0), NaCl (150 mM), 1% NP-40 (w/v), 10% glycerol (w/v), NaF (100 *μ*M), 1 mM phenylmethylsulphonyl fluoride (PMSF), aprotinin (20 *μ*g ml^–1^), sodium orthovanadate (1 mM; Na_3_VO_4_) and leupeptin (40 *μ*g ml^–1^). After incubation on ice for 30 min, cell lysates were centrifuged at 14 000 r.p.m. for 10 min at 4 °C and the supernatants were used for gel electrophoresis. After SDS–PAGE, the proteins were transferred to the PVDF membranes. Membranes were blocked with 5% non-fat dry milk in TBST (10 mM Tris-HCl, pH 8.0, 150 mM NaCl, 0.05% Tween20) and were then incubated with primary antibodies overnight at 4 °C. After washing five times for 10 min each in TBST, membranes were incubated with HRP-conjugated secondary antibodies for 1 h, washed again and the films were developed using a chemiluminescence method (ECL, PerkinElmer, Waltham, MA, USA).

### Chromatin immunoprecipitation experiments

Breast cancer cells were cross-linked with 1% formaldehyde in serum-free media at 37 °C for 10 min. Cells were then washed twice with ice-cold PBS and were resuspended with 300 *μ*l of lysis buffer (1% SDS, 10 mM EDTA, 50 mM Tris-HCl, pH 8.1, with protease inhibitor) and were incubated on ice for 10 min. Cell lysates were sonicated for three times at 10 s each and were centrifuged for 10 min at 14 000 r.p.m. at 4 °C. The supernatants were 1 : 10 diluted with CHIP dilution buffer (1% Triton X-100, 2 mM EDTA, 150 mM NaCl, 20 mM Tris-HCl, pH 8.1, 1 × protease inhibitor cocktail). In all, 100 *μ*l Dynal magnetic protein-G beads (Invitrogen) were washed twice with PBS plus BSA (5 mg ml^–1^) and were incubated with 5 *μ*g of either normal IgG or specific antibodies overnight at 4 °C. Beads were washed twice with PBS plus BSA (5 mg ml^–1^) and were incubated with 2 ml diluted chromatins at 4 °C overnight. Beads were then washed sequentially with 1 ml buffer1 (0.1% SDS, 1% Triton X-100, 2 mM EDTA, 20 mM Tris-HCl, pH 8.1, 150 mM NaCl), buffer2 (0.1% SDS, 1% Triton X-100, 2 mM EDTA, 20 mM Tris-HCl, pH 8.1, 500 mM NaCl), buffer3 (0.25 M LiCl, 1% NP-40, 1% deoxycholate, 1 mM EDTA, 10 mM Tris-HCl, pH 8.1) and twice with TE buffer. DNAs were eluted from the beads with 100 *μ*l of elution buffer (1% SDS, 0.1 M NaHCO_3_) at RT for 30 min. The products were then incubated at 65 °C for 8 h or overnight to reverse the cross-linking. DNAs were extracted using the QIAGEN PCR purification kit (Qiagen, Valencia, CA, USA). Real-time PCR was performed with the primers flanking the RBP-J*κ*-binding sites of human *HES1* promoter. The sequences of the primers used in CHIP and real-time PCR experiments are listed in Supplementary Table 1.

### Cell invasion assay

This assay was performed using a cell invasion kit from Cell Biolabs, Inc. (San Diego, CA, USA) Briefly, the invasion chambers were warmed up at room temperature for 10 min, and the basement membrane layer was rehydrated with 300 *μ*l of warm serum-free media for 1 h at room temperature. After removing the rehydration medium from the inserts, 300 *μ*l of 0.5–1.0 × 10^6^ cells ml^–1^ in serum-free media ± GSI was added to the inside of each insert, and 500 *μ*l of media containing 10% fetal bovine serum ± GSI was added to the lower well of the invasion plate. The plate was then incubated at either 21% O_2_ or 1% O_2_ for 48 h. The media was aspirated from the inside of the insert and the non-invasive cells were removed. The inserts were then transferred to clean wells containing 400 *μ*l of cell staining solution from the kit and were incubated for 10 min at room temperature. The stained inserts were washed several times with water and were air-dried. Inserts were transferred to new wells with 200 *μ*l of extraction solution from the kit and were incubated for 10 min with rotation. A volume of 100 *μ*l solution was used to measure the OD 560 nm in a spectrometer.

### Cell migration assay

This assay was performed using a kit from Cell Biolabs, Inc. All samples were tested in triplicates. Inserts were warmed up at room temperature for 10 min and their ‘wound field’ were aligned in the same direction. A cell suspension containing 0.5–1.0 × 10^6^ cells ml^–1^ in media containing 10% fetal bovine serum was generated. In all, 500 *μ*l of cell suspension was added to each well. Cells were cultured in the incubator until a monolayer formed. The inserts were then carefully removed from the wells, and the wells were washed with media to remove dead cells and debris. Finally, media ± GSI was added to the wells and the plates were incubated at either 21% O_2_ or 1% O_2_ for 48 h. The media was removed and 400 *μ*l of cell staining solution from the kit was added to each well and were incubated for 15 min at room temperature. The wells were washed three times with deionised water and were air-dried at room temperature.

## Results

### Notch signaling is active in human breast cancer cells

**Figure 1:**
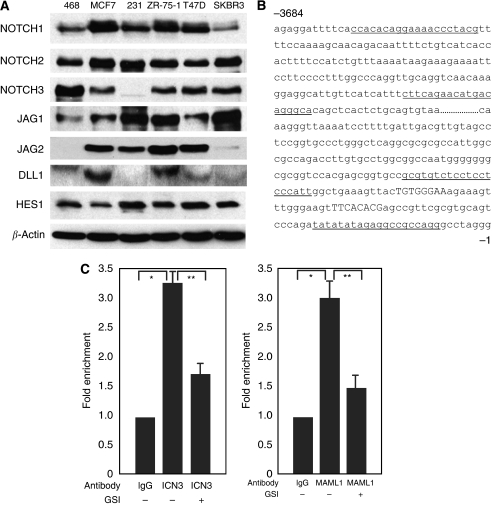
Notch signaling is active in human breast cancer cells. (**A**) Expression of Notch receptors, ligands and Notch target gene HES1 in breast cancer cells as detected by western blot analysis with specific antibodies. 468: MDA-468 cells; 231: MDA-231 cells. (**B**) Part of human *HES1* 5’ upstream sequence. The RBP-J*κ* binding sites are capitalised. The primer sequences flanking the RBP-J*κ* binding sites are underlined. The control primer sequences at approximately 3.7-kb upstream are also underlined. (**C**) Fold enrichment of the binding of NOTCH3 intracellular domain or MAML1 to the RBP-J*κ* binding sites of human *HES1* promoter in MCF7 cells with or without GSI treatment. Chromatin immunoprecipitation (CHIP) was performed with either normal IgG, ICN3-specific antibody or MAML1 antibody followed by real-time PCR with primers flanking the RBP-J*κ* binding sites of human *HES1* promoter. ^*^P<0.01; ^**^P<0.05

Western blot analysis showed that both NOTCH1 and NOTCH2 were expressed at different degrees in six human breast cancer cell lines (Figure 1A). NOTCH3 expression was not detectable in MDA-231 cells, but was detected in other five cell lines. There was no detectable expression of NOTCH4 in these cancer cells by either western blot analysis or RT–PCR (data not shown). As no gain-of-function mutations of Notch receptors have been found in breast cancer, ligand-mediated Notch activation still predominates in this context. We examined the expression of Notch ligands in these cancer cells and found that Notch ligand JAGGED1 was expressed at different degrees in these cancer cells (Figure 1A). JAGGED2 expression was detected in MCF7, MDA-231, T47D and ZR-75-1 cells, but was barely detectable in other two cell lines (Figure 1A). DELTA1 was expressed in MCF7, ZR-75-1 and T47D cells (Figure 1A). There was no detectable expression of DELTA3 and DELTA4 in these cancer cells (data not shown). The Notch target gene HES1 was also expressed at high levels in these cells (Figure 1A). Chromatin immunoprecipitation experiments showed that NOTCH3 intracellular domain and Notch co-activator MAML1 were present at the promoter of *HES1* gene in MCF7 cells (Figures 1B and C). Similar results were also obtained from MDA-468 cells (data not shown). *γ*-secretase inhibitor (GSI) treatment inhibited the binding of NOTCH3/MAML1 complex to *HES1* promoter (Figure 1C), indicating that Notch pathway is active in these breast cancer cells and MAML1 might be a co-activator of Notch signaling in breast cancer.

### Accumulation of HIF-1*α* and HIF-2*α* in breast cancer cells with hypoxia

**Figure 2:**
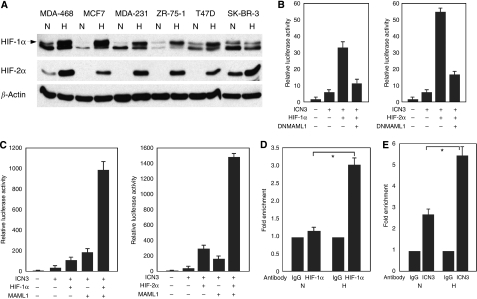
Hypoxia inducible factor (HIF)-1*α* and HIF-2*α* accumulated in breast cancer cells with hypoxia and they potentiated Notch signaling. (**A**) Western blot analysis of HIF-1*α* (the upper band marked by the arrow) and HIF-2*α* expression in breast cancer cells under 21% O_2_ or 1% O_2_ culture conditions for 24 h. H, hypoxia; N, normoxia. (**B**) Enhanced ICN3 signaling by HIF-1*α* or HIF-2*α* as determined by an *in vitro* Notch reporter assay, which was inhibited by dominant negative MAML1. (**C**) Hypoxia inducible factor (HIF)-1*α* and HIF-2*α* synergise with MAML1 in potentiating ICN3-mediated Notch signaling. (**D**) Enrichment of HIF-1*α* at *HES1* promoter by hypoxia in MCF7 cells. Fold enrichment of the binding of HIF-1*α* to the RBP-J*κ* binding sites of human *HES1* promoter in MCF7 cells under 1% O_2_ culture condition. Normal IgG or HIF-1*α*-specific antibodies were used for CHIP experiments followed by real-time PCR with primers flanking the RBP-J*κ* binding sites of *HES1* promoter. H, hypoxia; N, normoxia; ^*^P<0.01. (**E**) Increased binding of NOTCH3 intracellular domain to the RBP-J*κ* binding sites of *HES1* promoter in MCF7 cells under hypoxia. H: hypoxia; N, normoxia; ^*^P<0.05.

Hypoxia inducible factors are heterodimeric proteins that belong to the basic helix-loop-helix transcription factor families. Hypoxia inducible factor-1*α* was expressed at a low level in MDA-231, MDA-468, T47D, SK-BR-3 and ZR-75-1 cells under normal culture conditions (Figure 2A). However, it dramatically accumulated in these cells when they were cultured under hypoxia (Figure 2A), except for SK-BR-3 cells, which have a higher basal level HIF-1*α* expression. Hypoxia inducible factor-2*α* expression was only detected at different degrees in MDA-468 and SK-BR-3 cells, but not in other cell lines when they were cultured at regular culture conditions. However, HIF-2*α* also dramatically accumulated in these cells with hypoxia (Figure 2A).

### HIF-1*α* and HIF-2*α* potentiated Notch signaling

Using an *in vitro* Notch reporter assay, we tested the effects of HIF-1*α* and HIF-2*α* on Notch signaling. Both HIF-1*α* and HIF-2*α* potentiated ICN1- and ICN3-induced Notch signaling, with HIF-2*α* being more potent (Figure 2B and data not shown). Dominant negative MAML1 (DNMAML1) can abrogate the effects of HIF factors on Notch signaling, implicating MAML1 as a key co-activator of this Notch activation complex (Figure 2B). Interestingly, co-transfection of HIF-1*α* or HIF-2*α* with full-length MAML1 into cancer cells induced ICN3-mediated Notch signaling dramatically (Figure 2C), indicating a strong synergy between HIF factors and MAML1 in the activation of Notch pathway.

### Enrichment of HIF-1*α* and Notch intracellular domain at *HES1* promoter with hypoxia

To study the mechanisms whereby HIF-1*α* and HIF-2*α* augmented Notch signaling in breast cancer cells, CHIP experiments with a HIF-1*α* antibody in MCF7 cells were performed. Within human *HES1* promoter sequence, there are two RBP-J*κ* binding sites that are approximately 16 bp apart. Hypoxia inducible factor-1*α* was enriched at these sites approximately three-folds after hypoxia (Figure 2D). As there is no direct HIF-1*α* binding site within this part of *HES1* promoter, HIF-1*α* was likely enriched through its binding to the intracellular domain of Notch receptors (Gustafsson *et al*, 2005). The binding of NOTCH3 intracellular domain to *HES1* promoter was also increased in MCF7 cells when they were cultured under 1% O_2_ condition (Figure 2E).

### Increased expression of Notch target genes in breast cancer cells with hypoxia

**Figure 3:**
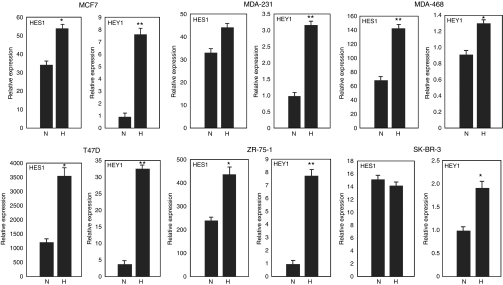
Increased Notch target gene expression in breast cancer cells by hypoxia. Real-time PCR analysis of the expression of Notch target genes *HES1* and *HEY1* in breast cancer cells under 1% O_2_ culture condition for 24 h. For MCF7 cells: ^*^P<0.05, ^**^P<0.001. For MDA-231 cells: ^**^P<0.01. For MDA-468 cells: ^*^P<0.05, ^**^P<0.01. For T47D cells: ^*^P<0.01, ^**^P<0.001. For ZR-75-1 cells: ^*^P<0.05, ^**^P<0.001. For SK-BR-3 cells: ^*^P<0.05. H, hypoxia; N, normoxia.

We then examined the expression of Notch target genes in these breast cancer cells when they were cultured at either normoxic or hypoxic conditions. The expression of Notch target genes such as *HES1* and *HEY1* were increased in MCF7 cells and MDA-468 cells when they were cultured at 1% O_2_ condition (Figure 3), indicating that hypoxia, presumably through induction of HIF factor expression, regulates Notch signaling in breast cancer cells. Culture of other breast cancer cells under hypoxia also increased the expression of *HES1* and *HEY1* to different degrees (Figure 3), except for SK-BR-3 cells, which did not have an elevated *HES1* expression under hypoxic conditions.

*HES1* was highly expressed in these cancer cells under normal culture conditions. However, the increase of *HES1* expression under hypoxia was not dramatic. *HEY1* expression was at a very low level in these cancer cells under normoxia, and was greatly induced in these cells with hypoxia, indicating *HEY1* gene is an excellent potential marker of Notch pathway activation in human breast cancer cell lines.

**Figure 4:**
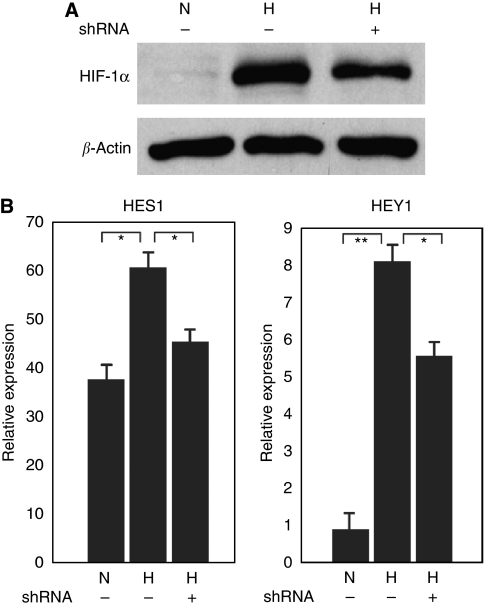
The increased expression of *HES1* and *HEY1* in MCF7 cells under hypoxia is HIF-1*α* dependant. (**A**) Western blot analysis of HIF-1*α* expression in MCF7 cells under hypoxia with the knockdown of *HIF-1α* by shRNA. (**B**) Real-time PCR analysis of *HES1* and *HEY1* expression in MCF7 cells under hypoxia with the knockdown of *HIF-1α* by shRNA. ^*^P<0.05, ^**^P<0.001. H, hypoxia; N, normoxia.

To study whether the effect of hypoxia on *HES1* and *HEY1* expression is mediated by HIF transcription factors, we knocked down *HIF-1α* expression with shRNA and found that both *HES1* and *HEY1* expression were inhibited (Figure 4), indicating that their increased expression under hypoxia is HIF-1*α* dependant.

**Figure 5:**
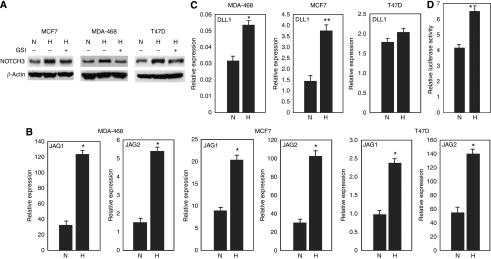
Increased Notch receptor and Notch ligand expression in breast cancer cells by hypoxia. (**A**) Western blot analysis of the expression of NOTCH3 in breast cancer cells under 21% O_2_ or 1% O_2_ culture conditions with or without GSI treatment. H, hypoxia; N, normoxia. (**B**) Expression of Notch ligands *JAGGED1* and *JAGGED2* in breast cancer cells under hypoxia. ^*^P<0.01. (**C**) Expression of Notch ligand *DELTA1* in breast cancer cells under hypoxia. ^*^P<0.05; ^**^P<0.01. (**D**) MCF7 cells were transfected with *HES1-luc* and *TK-Renilla* luciferase reporter genes and were cultured at 21% or 1% O_2_ conditions. Dual-luciferase assays were performed 48 h after transfection. ^*^P<0.05; H, hypoxia; N, normoxia.

We also tested the protein levels of Notch receptors in these cells when they were cultured at low oxygen concentration. Hypoxia increased NOTCH3 expression in MDA-468, MCF7 and T47D cells and this increase was abrogated by GSI treatment (Figure 5A). In addition to NOTCH3, the expression of Notch ligands *JAGGED1* and *JAGGED2* was also increased in these cells by hypoxia (Figure 5B). *DELTA1* expression was also elevated in MCF7 cells and MDA-468 cells by hypoxia (Figure 5C). Finally, elevated *HES1*-luc reporter gene activity was detected in the transfected MCF7 cells when they were cultured at 1% O_2_ condition (Figure 5D).

### Altered expression of Slug, Snail and E-cadherin in breast cancer cells with hypoxia

**Figure 6:**
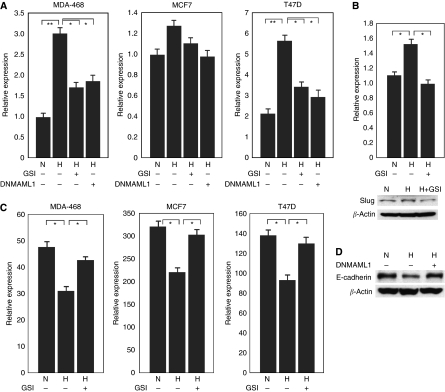
Altered expression of Slug, Snail and E-cadherin in breast cancer cells by hypoxia. (**A**) Real-time PCR analysis of the expression of *Snail* in breast cancer cells with hypoxia. The increased expression of *Snail* with hypoxia was abrogated by either GSI treatment or DNMAML1. ^*^P<0.05; ^**^P<0.01. (**B**) Slug expression (both mRNA level and protein level) was increased in MDA-231 cells with hypoxia, which was abrogated by GSI treatment. ^*^P<0.05. (**C**) Real-time PCR analysis of the expression of *E-cadherin* in breast cancer cells under 21% O_2_ or 1% O_2_ culture conditions with or without GSI treatment. ^*^P<0.05. (**D**) Western blot analysis of E-cadherin expression in MCF7 cells with hypoxia. Dominant negative MAML1 abrogated the downregulation of E-cadherin during hypoxia. H, hypoxia; N, normoxia.

Zinc-finger proteins Slug and Snail are two known E-cadherin repressors that initiate EMT in breast cancer. The expression of Slug and Snail has been shown to be regulated by Notch pathway. *Snail* expression was increased significantly in MDA-468 and T47D breast cancer cells with hypoxia, and this increase was abrogated by GSI treatment (Figure 6A). Inhibition of Notch signaling by DNMAML1 in MDA-468 cells and T47D cells also abrogated hypoxia-mediated *Snail* expression (Figure 6A). *Snail* expression was also slightly increased in MCF7 cells with hypoxia. Slug is only expressed in MDA-231 cells under regular culture conditions. With hypoxia, Slug expression was increased in MDA-231 cells (Figure 6B), and this increased expression was also abrogated by GSI treatment (Figure 6B).

E-cadherin is a cell–cell adhesion molecule that participates in calcium-dependant interactions to form epithelial adherens junctions. Downregulation of E-cadherin is one of the best markers of EMT in human breast cancer. *E-cadherin* was expressed at different degrees in MDA-468, MCF7 and T47D cells (Figure 6C), but was not expressed in MDA-231 and SK-BR-3 cells (data not shown). Under hypoxia, the mRNA levels of *E-cadherin* were reduced in MDA-468, MCF7 and T47D cells (Figure 6C). The GSI treatment abrogated the downregulation of *E-cadherin* by hypoxia in these cell lines (Figure 6C). E-cadherin protein levels were also decreased in MCF7 cells when they were cultured under hypoxic conditions (Figure 6D). Inhibition of Notch signaling by DNMAML1 abrogated the downregulation of E-cadherin observed during hypoxia in MCF7 cells (Figure 6D).

### Notch signaling is required for hypoxia-mediated breast cancer cell migration and invasion

**Figure 7:**
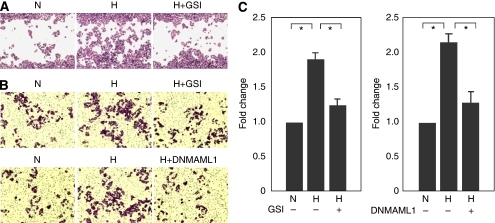
Notch signaling is required for hypoxia-mediated MDA-468 cell migration and invasion. (**A**) The closure of scratch wound of MDA-468 cells was facilitated by hypoxia and was inhibited by GSI treatment. (**B** and **C**) The migration of MDA-468 breast cancer cells across a basement membrane toward the serum attraction was elevated by hypoxia and was abrogated by either GSI treatment or DNMAML1. ^*^P<0.05; H, hypoxia; N, normoxia.

Increased cell migration and invasion are important consequences of hypoxia-induced EMT. To study the effects of hypoxia on breast cancer cell motility and invasiveness, and whether Notch signaling is required for these effects, breast cancer cells from 21 or 1% O_2_ culture conditions with or without the GSI treatment were applied to a scratch wound-healing assay. Culture of MDA-468 cells under hypoxia increased the closure of the scratch wound compared with the control (Figure 7A). The GSI treatment inhibited the migration of MDA-468 cells across the scratch wound (Figure 7A). To study the invasive properties of these breast cancer cells under normoxia or hypoxia, a basement membrane invasion assay was performed. Culture of MDA-468 cells at 1% O_2_ concentration increased the migration of these cells across a membrane toward a source of serum attraction (Figures 7B and C). The GSI treatment of MDA-468 cells or transfection of DNMAML1 inhibited the migration of these cells across the membrane (Figures 7B and C), indicating that Notch pathway is required for hypoxia-mediated breast cancer cell migration and invasion.

## Discussion

Our study showed that both HIF-1*α* and HIF-2*α* accumulated in breast cancer cells with hypoxia and they potentiated Notch signaling. Notch target gene expression was induced in these cells when they were cultured at low oxygen conditions. shRNA-mediated knockdown of HIF-1*α* abrogated hypoxia-induced *HES1* and *HEY1* expression, indicating the effect of hypoxia on Notch activation is via HIF factors. Interestingly, HIF-1*α* and HIF-2*α* synergised with MAML1 in the activation of Notch pathway in the *in vitro* Notch reporter assay and dominant negative MAML1 can block HIF-induced Notch signaling, indicating MAML1 as a key co-activator among the Notch activation complex. MAML1 has been detected earlier as part of a Notch/HIF/MAML1 immuno-complex (Sahlgren *et al*, 2008). We did not detect a direct interaction between HIF factors and full length MAML1 or RBP-J*κ* (J Chen and J Griffin, unpublished data). Therefore, MAML1 and HIF factors may form a complex with Notch intracellular domain and function as scaffold proteins to recruit CBP/p300 and other Notch co-activators to the whole complex.

SK-BR-3 cells have a higher basal level expression of both HIF-1*α* and HIF-2*α* compared with other breast cancer cells. Under hypoxia, the expression of HIF-1*α* in these cells was not increased while the expression of HIF-2*α* was slightly elevated. This might be the reason why there was only two-fold increase of *HEY1* expression and no elevated *HES1* expression in these cells with hypoxia.

To date, cell autonomous gain-of-function mutations in Notch receptors have not been reported in human breast cancers and other solid tumours, suggesting that ligand-mediated Notch activation predominates in these contexts. We detected the expression of Notch ligands JAGGED1, JAGGED2 and DELTA1 in these breast cancer cells. They might have a role in the stimulation of Notch pathway in these cells. Elevated NOTCH3 expression was detected in MCF7, MDA-468 and T47D cells after hypoxia, and the expression of *JAGGED1*, *JAGGED2* and *DELTA1* ligands was also increased in these cells after they were cultured at 1% O_2_ condition. This indicated that increased ligand levels and stabilisation of Notch receptors are likely to act together to increase Notch signaling under hypoxia. Furthermore, our studies suggested that hypoxia not only potentiate the pre-existing Notch signaling in breast cancer cells, it also induce Notch signaling by increasing the expression of both Notch receptors and ligands.

Our study found that the expression of Notch target genes *HES1* and *HEY1* was increased in most breast cancer cells with hypoxia. However, the increased *HEY1* expression by hypoxia was more dramatic compared with *HES1* expression, indicating *HEY1* might be a better marker of Notch activation in breast cancer cells. Previous studies also showed that *HEY* genes are the most sensitive Notch target genes for Notch pathway inhibition in breast cancer (Leong *et al*, 2007). Positive expression correlations between *JAGGED1* and *HEY* genes have also been identified in primary human breast cancer (Leong *et al*, 2007).

Epithelial-to-mesenchymal transition is characterised by loss of cell adhesion, repression of E-cadherin expression and increased cell mobility. Induction of EMT in immortalised human mammary epithelial cells results in the expression of stem cell markers (Mani *et al*, 2008). Several oncogenic pathways such as TGF-*β*, receptor tyrosine kinases (RTKs), integrin, endothelin A receptor, Wnt/beta-catenin, hypoxia, matrix metalloproteinase (MMPs) and Notch induce EMT (Polyak and Weinberg, 2009). Downregulation of E-cadherin is one of the best markers of EMT in breast cancers. Zinc-finger proteins Snail and Slug are two transcriptional repressors of E-cadherin and their expression induces EMT and promotes malignant cell invasion and dissemination. Other E-cadherin transcriptional repressors include SIP1, TWIST1, FOXC2, FOXC1 and ZEB1 (Polyak and Weinberg, 2009). Some non-coding microRNAs have also been implicated as regulators of EMT and tumour metastasis (Ma *et al*, 2007; Gregory *et al*, 2008; Park *et al*, 2008; Tavazoie *et al*, 2008).

Previous studies have suggested that Notch signaling induces a particular type of EMT during normal heart development and that Notch increases Snail expression in endothelial cells to promote mesenchymal transformation (Noseda *et al*, 2004; Timmerman *et al*, 2004). Recent studies showed that JAGGED1-induced Notch signaling induces EMT through Slug-mediated repression of E-cadherin (Leong *et al*, 2007). Over-expression of NOTCH1 intracellular domain, but not *HES1* or *HEY1*, inhibits E-cadherin expression and induces EMT in ovarian cancer cells, probably through the induction of Snail expression (Sahlgren *et al*, 2008). Our studies indicate that hypoxia may be an initiative event resulting in enhanced Notch signaling, increasing the expression of Slug and Snail in breast cancer cells, which in turn inhibited E-cadherin expression. Inhibition of Notch signaling by dominant negative MAML1 abrogated the downregulation of E-cadherin observed during hypoxia. Culture of breast cancer cells under hypoxia increased their migration and invasion, which is abolished by Notch pathway inhibition with either GSI treatment or DNMAML1. These studies suggested that Notch signaling is not only required for the proliferation of breast cancer cells under regular culture conditions, it is also required, under hypoxia, to further activate Notch signaling, to initiate EMT, and thereby increasing the likelihood of increased breast cancer cell migration, invasion and metastasis. Over the short term, pharmacological inhibitors of ligand-induced Notch signaling might be able to block EMT and tumour metastasis in the treatment of human breast cancer.
