# Supplementary material for: Hypoxia potentiates Notch signaling in breast cancer leading to decreased E-cadherin expression and increased cell migration and invasion
Source: Br J Cancer. 2009 Dec 15;102(2):351–60. doi: 10.1038/sj.bjc.6605486 (PMC2816657; doi:10.1038/sj.bjc.6605486)
Supplement: Supplementary Table [file 6605486x1.doc]

**Supplementary Materials**

Table 1. Primers for real-time PCR and CHIP experiments

| **genes** | **forward primer** | **reverse primer** |
| --- | --- | --- |
| control primers  for CHIP | 5’-CCACACAGGAAAACCCTACG-3’ | 5’-TGCCCTGTCATGTTCTGAAG-3’ |
| primers for  *HES1* promoter | 5’-GCGTGTCTCCTCCTCCCATT-3’ | 5’-CCTGGCGGCCTCTATATATA-3’ |
| *β-actin* | 5’-TCCCTGGAGAAGAGCTACGA-3’ | 5’-AGCACTGTGTTGGCGTACAG-3’ |
| *HES1* | 5’-TCAACACGACACCGGATAAA-3’ | 5’-CCGCGAGCTATCTTTCTTCA-3’ |
| *HEY1* | 5’-CGAGGTGGAGAAGGAGAGTG-3’ | 5’-CTGGGTACCAGCCTTCTCAG-3’ |
| *E-cadherin* | 5’-CAGCACGTACACAGCCCTAA-3’ | 5’-ACCTGAGGCTTTGGATTCCT-3’ |
| *SLUG* | 5’-AGATGCATATTCGGACCCAC-3’ | 5’-CCTCATGTTTGTGCAGGAGA-3’ |
| *SNAIL* | 5’-CTTCCAGCAGCCCTACGAC-3’ | 5’-CGGTGGGGTTGAGGATCT-3’ |
| *DELTA1* | 5’-TGCAACCCTGGCTGGAAA-3’ | 5’-AATCCATGCTGCTCATCACATC-3’ |
| *JAGGED1* | 5’-GACTCATCAGCCGTGTCTCA-3’ | 5’-TGGGGAACACTCACACTCAA-3’ |
| *JAGGED2* | 5’-TCTGCCTTGCTACAATGGTG-3’ | 5’-GCGATACCCGTTGATCTCAT-3’ |
| *NOTCH1* | 5’-CACTGTGGGCGGGTCC-3’ | 5’-GTTGTATTGGTTCGGCACCAT-3’ |
| *NOTCH2* | 5’-AATCCCTGACTCCAGAACG-3’ | 5’-TGGTAGACCAAGTCTGTGATGAT-3’ |
| *NOTCH4* | 5’-TAGGGCTCCCCAGCTCTC-3’ | 5’-GGCAGGTGCCCCCATT-3’ |
